# Optimization and interlaboratory validation of the HydroTouch test for fingertip-mediated microbial transmission and antimicrobial efficacy assessment of surfaces

**DOI:** 10.1186/s12866-026-05312-0

**Published:** 2026-06-25

**Authors:** Roberto B.M. Marano, Mihyun Lee, Celeste Peterson, Brenda Raid, Mila S. Wolters, Niclas Nordholt, Susann Wiltner, Luzia Wiesli, Mati Kook, Henryk Schnaars, Dagmar K. Willkomm, Qun Ren, Frank Schreiber, Angela Ivask

**Affiliations:** 1 Division Biodeterioration and Reference Organisms, Department of Materials and the Environment, Federal Institute for Materials Research and Testing, Berlin, 12205 Germany; 2 Laboratory for Biointerfaces, Empa, Swiss Federal Laboratories for Materials Science and Technology, Lerchenfeldstrasse 5, St. Gallen, 9014 Switzerland; 3 Institute of Molecular and Cell Biology, University of Tartu, Riia 23, Tartu, 51010 Estonia; 4 Drägerwerk AG & Co. KGaA, Moislinger Allee 53-55, Lübeck, 23558 Germany; 5 Institute of Physics, University of Tartu, Ostwaldi 1, Tartu, 50411 Estonia; 6 Technische Hochschule Lübeck, Mönkhofer Weg 239, Lübeck, 23562 Germany

**Keywords:** Antimicrobial surfaces, Antimicrobial materials, Copper, Antimicrobial coatings, Transfer of pathogens, Biocidal product regulation, Treated surfaces, Soiling, Standardization

## Abstract

**Background:**

Surface-mediated transmission of microorganisms may lead to severe healthcare or food industry related outbreaks, and antimicrobial surfaces are one of the means to stop or decrease the surface transfer of bacteria. Standardized test methods conducted under idealized conditions and traditionally used to assess the antimicrobial activity of such surfaces do not however accurately reflect the real-life conditions of surfaces used in dry environments and high-touch settings. Previously, we have designed the HydroTouch test, which uses a semi-dry microbial inoculum on a finger-mimetic hydrogel to enable reliable quantification of microbial transfer efficiency under real-use-like conditions on environmental surfaces. Here, the HydroTouch test was refined in terms of microbial inoculum density, post-transfer exposure time and environmental conditions during surface exposure to enable the determination of surface transfer and antimicrobial activity.

**Results:**

By using metallic copper as a model antimicrobial material, we showed that exposure time after microbial transfer from the finger-mimetic hydrogel to the surface is critical: short contact periods result in insufficient antimicrobial activity, whereas prolonged exposure leads to increased surface kill over time due to desiccation, complicating the interpretation of the antimicrobial effect. Among four bacteria and yeast tested, only *Escherichia coli* and *Staphylococcus aureus* showed sufficient surface transfer and viability on controls to enable their reliable use on the HydroTouch test. Organic soiling during surface exposure was shown to have notably enhanced surface transfer of bacteria but at the same time it also reduced the activity of copper surface. Interlaboratory comparison of the method carried out in four laboratories indicated 3–17% bacterial surface transfer in different labs, whereas the interlaboratory differences could be attributed to variability in relative air humidity during touch surface preparation and exposure. During 10 min exposure a modest but significant reduction of 0.30–0.75 log_10_ in viable cell count on copper was observed by the participating laboratories and also this parameter was clearly affected by humidity of the exposure environment.

**Conclusions:**

The utility of the modified HydroTouch test for the determination of microbial surface transfer and antimicrobial activity across different laboratories was demonstated with organic soiling and air humidity being the decisive factors for its reproducibility.

## Introduction

 The importance of good hygiene practices and control of microbial contamination is receiving ever more attention. An important pathway for the transfer of microbial contaminants is through environmental surfaces, which can act as reservoirs of microorganisms. These surfaces become contaminated either from natural sources or via human carriers, and microbial cells can survive on them for extended periods [1–3]. The importance of surface-mediated transfer of microbial contaminants between humans has been described in common areas [4, 5], in domestic environment [6], in food industry [7] and most importantly in hospitals [4, 8, 9] where contact transmission of potentially pathogenic microorganisms may be responsible for severe medical outbreaks [10, 11]. Strategies to reduce the potential for microbial transfer via surfaces include cyclic cleaning and disinfection as well as deployment of surfaces with persistent antimicrobial effect, the popularity of which shows an increasing trend [12, 13].

Currently, the activity of antimicrobial surface coating products is evaluated for commercialization primarily through standardized testing methods prior to market approval. For antimicrobial touch surfaces, efficacy is most commonly assessed using ISO 22196:2011 [14], while photocatalytic surfaces are evaluated using ISO 27447:2019 [15]. These methods support antimicrobial claims under the EU Biocidal Products regulation [16] but rely on moist, liquid-based conditions that poorly reflect the typically semi-dry or dry environments of high-touch surfaces. Consequently, ISO‑measured efficacy may not accurately predict real‑world transmission‑reducing performance.

Several studies have examined the impact of different antimicrobial test formats and conditions, including variations in relative humidity, and have consistently reported substantial variability in efficacy outcomes. Wiegand et al. [17] and Campos et al. [18] showed that most of the interlaboratory variation in antimicrobial efficacy could arise from incubation‑chamber humidity and organic matter. Similarly Kaur et al. [19] demonstrated that antibacterial effects of copper, silver and quaternary ammonium-based surfaces diminished under low humidity and rapid drying conditions and the same has been shown for an antimicrobial thin film [17] and a zinc-based surface [19]. This suggests that antimicrobial surface efficacy is highly test‑condition dependent, underscoring the importance of selecting methods aligned with the intended application.

While all the above discussed antimicrobial efficacy test methods are based on direct application of microbial cells in liquid form onto test surfaces, they do not involve surface touch, which is a primary route of microbial transfer to and from relevant human-exposed surfaces. To address this, a range of touch-based methods has been developed [20–22]. These methods vary in details, but generally involve transferring microorganisms to surfaces either through direct finger contact or via a donor surface. A major challenge in quantifying the antimicrobial activity of surfaces using conventional dry-touch transfer tests is that bacteria experience stress during the drying process due to desiccation and increasing salinity conditions [23]. These factors can affect microbial viability on the surface, thereby influencing the measured antimicrobial activity. For example, Campos et al. [18] and Knobloch et al. [24] demonstrated that traditionally active silver surface failed to have an antimicrobial effect on bacteria in a dry-touch transfer test. Mayr et al. [25] reported on a similar finding for a previously known antimicrobial surface in an interlaboratory study. Additionally, microbial transfer efficiency—the ratio of bacteria transferred to the test surface relative to the initial number on the donor surface—cannot be accurately determined because of the decreased bacterial viability after drying. To overcome these limitations and accurately assess microbial transmission, we previously developed the HydroTouch test, which uses a semi-dry inoculum on a gelatin hydrogel as the donor surface to mimic human fingers [26]. We demonstrated that the semi-dry environment of the gelatin hydrogel preserves microbial viability during a preincubation step that involves limited loss of moisture. Furthermore, the number of viable bacteria remaining on the hydrogel after pre-incubation and touch transfer can be precisely quantified by exploiting the temperature-induced sol–gel transition of the gelatin, enabling accurate calculation of live bacterial transfer efficiency onto various test surfaces.

In this study, we further refined the HydroTouch testing method to accurately evaluate the antimicrobial activity of surfaces under relevant conditions. We systematically examined how experimental factors, such as inoculum size, exposure time on the test surface after touch, and the presence of soiling, affect bacterial surface transfer, and optimized conditions for reliable detection of antimicrobial activity of surface samples by measuring the reduction of viable cells over time. Furthermore, we conducted an interlaboratory testing across four laboratories to demonstrate the reproducibility of the HydroTouch method, using both a non-antimicrobial surface (stainless steel) and a benchmark antimicrobial surface (metallic copper). We envision that the HydroTouch test, with the parameters optimized in this study, will provide a reliable quantitative and standardizable approach for assessing the antimicrobial efficacy of various environmental surfaces under a real-life mimicking condition.

## Materials and methods

### Microbial strains and their preparation for the HydroTouch test

For the HydroTouch test, four bacteria (*E. coli* ATCC 8739, *S. aureus* ATCC 6538P, * Pseudomonas aeruginosa* ATCC 15442, *Acinetobacter baumannii* ATCC 17978) and one yeast (*Candida albicans* ATCC 90028) were used. Those microbial species were selected due to their common use in the laboratory and relevance in clinical environment, and the strains were selected to match the recommendations of existing antimicrobial testing standards. To prepare the bacterial strains, a bacterial colony was transferred to a plate count agar (PCA) plate and incubated overnight at 37 °C. After incubation, the colonies were transferred from the PCA plates into 5 mL of 30% tryptic soy broth (TSB; Neogen NCM0019A) with 0.25% glucose to a cell density of 10^3^ CFU/mL. The tube was incubated 24 ± 1 h at 37 °C at 160 rpm. Then, OD_600_ was measured and the culture was diluted with 1:500 diluted nutrient broth (NB; per litre: 3.0 g of meat extract, 10.0 g of peptone, and 5.0 g of sodium chloride) until the desired cell density was reached. The used OD_600_ values that resulted in 1 × 10^6^ cells in 5 µL, as was used in the HydroTouch test, were: 0.7 for *E. coli*, 1.0 for *S. aureus*, 0.4 for *P. aeruginosa*,* and* 0.5 for *A. baumannii*. For *E. coli* three cell densities were used: OD_600_ of 0.04, which resulted in 5 × 10^4^ cells in 5 µL, OD_600_ of 0.08, which resulted in 1 × 10^5^ cells in 5 µL, and OD_600_ of 0.7, which resulted in 1 × 10^6^ cells in 5 µL. The relationship between OD600 and cell number was applied uniformly across all laboratories. In some assays, an artificial test soil (ATS) consisting of 33% of proteins, 11% of carbohydrates, and minor quantities of cellulose, lipids, mucin and other organic constituents (Healthmark Industries Co, product ATS2015 without added defibrinated sheep blood), was added in 1:1 ratio to double concentrated bacterial inoculum either undiluted (37 mg ATS/mL, the suggested soiling concentration by the producer) or after diluting it 1:5 (7.4 mg ATS/mL) with 1:500 diluted nutrient broth similarly to what was previously described by Marano et al. [27]. Therefore, the final ATS concentrations in the test were 18 and 3.7 mg/mL. To prepare yeast *C. albicans*, an overnight colony on a fresh YPD agar plate (per litre: 10 g yeast extract, 20 g peptone, 20 g dextrose, 60 g agar) was transferred to fresh liquid YPD medium at a cell density of 10^3^ CFU/mL, the tube was incubated for 24 ± 1 h at 37 °C at 160 rpm, the cells were centrifuged and finally resuspended in 1:500 NB medium to an OD_600_ of 30 equivalent that translated to 1 × 10^6^ cells in 5 µL as was used in the HydroTouch test.

### Surfaces used in the HydroTouch test

The transfer of microorganisms during the HydroTouch test was carried out onto cold-rolled, mirror-polished AISI 304–304 L/DIN1.4301-1.4307 stainless steel plates (SS; Cr-18–20%, Ni-8–10.5%), and ≥ 99.9% purity copper surfaces [Copper alloy Cu-DHP (EN 1412: CW024A, UNS C12200) supplied in temper R360 (extra hard, cold-worked), supplied by Wieland Group, Ulm, Germany]. The metal surfaces were cut to 1 cm^2^ squares, which were washed with 70% ethanol and then dried under a biosafety cabinet prior to the HydroTouch test. Scanning electron microscopy images of the surfaces were recorded using a digital optical microscope (Euromex sCMEX-32). Contact angles were measured according to the earlier published protocol [28] by taking images of six 1 µl drops of water on each surface and analysing the water contact angles using ImageJ with the LBADSA plugin.

### HydroTouch method

**Fig. 1:**
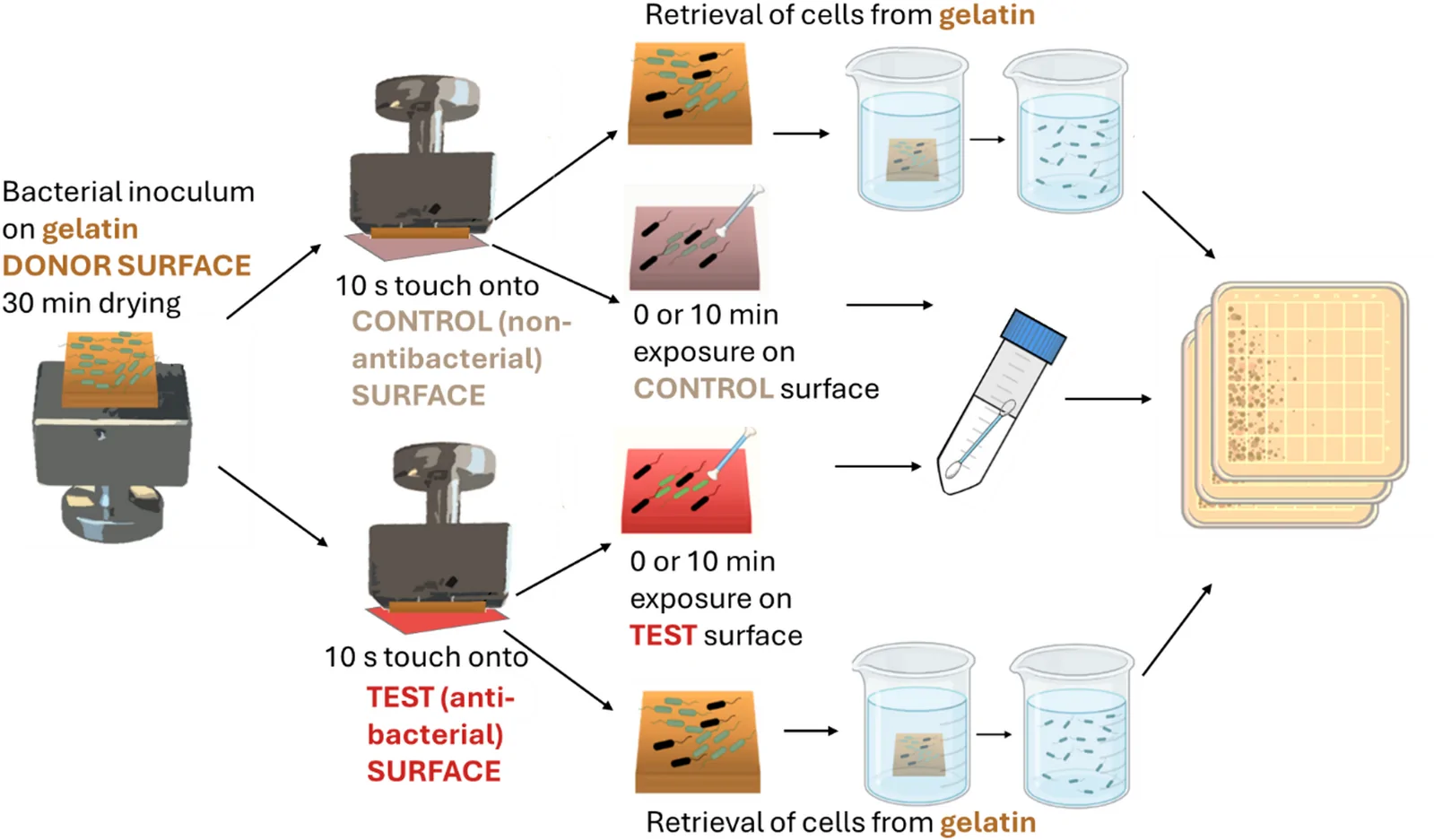
Schematics of HydroTouch test. The critical steps of the method are shown and designation of the surfaces is indicated. Figure is modified from [26]

The basic HydroTouch test method has been described by us earlier [26], however several adjustments were introduced in this study (see the general test scheme on Fig. 1). Preparation for the test involved preparation of a 10% suspension of gelatin (gelatin from pork skin Sigma-Aldrich, G2500), dissolution of the gelatin in distilled water using a magnetic stirrer at 60 °C for 20 min and immediate filtering through a 0.45 μm microfilter. 22 mL of filtered gelatin was poured to a 10 cm diameter Petri dish and solidified at room temperature on a sterile bench for 60 min. The plates were then kept at 4 °C until use (within 24 h). The herein described hands-on operations were conducted in a sterile bench. Before the test, 1.1 × 1.1 cm squared pieces were cut from the gelatin using a custom-made cutting device as described in [26]. Gelatin pieces were then placed onto metal holders (weight of each holder 50 g) and 5 µL of bacterial or yeast inoculum in previously described media was added to the surface by spreading it with an inoculation loop. These gelatin pads, devised to mimic a human fingertip carrying bacteria, were then incubated in a closed box (to preserve moisture) at room temperature for 30 min (except in one lab that during interlaboratory screening incubated the gelatin in an open Petri container) and then touched during 10 s to test (recipient) surfaces that had been previously placed to PDMS (PDMS Silicone Elastomer Kit, Dow Corning) containing Petri dishes to help hold them steady. For touching, only the weight of the metal holder (50 g) was used, no additional force was applied. After detaching, the gelatin pads from recipient surfaces these were placed to 2 mL phosphate-buffered saline (PBS) supplemented with neutralizer (per 100 mL PBS, 0.1 g of lecithin and 0.7 mL Polysorbate 80) on a 12-well plate. For quantification of the initial number of colony forming units loaded onto gelatin pads, a gelatin pad with dried bacterial or yeast inoculum but not touched to the surface was also placed into the solution in the 12-well plate. The 12-well plate was then incubated at 37 °C for 20 to 30 min, shaking at 160 rpm, until gelatine was fully dissolved. Serial dilutions were prepared with PBS, and 20 µL of each dilution was spotted to PCA (bacteria) or YPD agar plates (yeast) in triplicate, followed by overnight growth at 37 °C and colony counting. From touched surfaces, the residing microorganisms were swabbed using double-tipped cotton swabs immediately after touching (in practice ca. 20 s after touch, referred to as 0 min incubation), and 10 min, 30–60 min after touching. During incubation and before swabbing, the surfaces were stored in a closed box at room temperature (except laboratory 4 participating in the interlaboratory trial, having the surfaces under the sterile bench for the 10 min incubation). Importantly, laboratory 3 participating in the interlaboratory trial incubated each gelatin pad with bacteria and each recipient surface in a separate box, thus creating slightly higher relative humidity during the test than other laboratories. All the laboratories participating in the interlaboratory trial registered the relative air humidity during their respective exposure conditions. For cell recovery from recipient surfaces, one head of a two headed common hygiene cotton swab was pre-wetted with 100 µL soy casein digest lecithin polysorbate broth (SCDLP; per liter: 17 g tryptone, 3 g soy peptone, 5 g NaCl, 2.5 g glucose, 2.5 g K_2_HPO_4_, 1 g lecithin, 7 g polysorbate 80) and used to swab the surface first, followed by a second swabbing with the other dry head. Both cotton swab tips were cut with sterile scissors and collected in 2 mL SCLDP-containing tubes. The tubes were vortexed for 30 s and serial dilutions were performed in PBS. 20 µL of each dilution was spotted on PCA (bacteria) or YPD agar plates (yeast) in triplicate. In addition, 500 µL of the wash-off was plated on PCA or YPD agar plates. Plates were incubated overnight at 37 °C, followed by CFU counting. CFU counts on gelatin and on surfaces were log converted (log_10_). The limit of the detection of the transfer test was 1.2 log_10_ CFU (16 CFU), which was calculated by considering 3 CFU as the minimal countable number of CFUs and taking into account the dilution factor. The dynamic range of measureable CFUs number in the system was therefore 1.2-6 log_10_.

### Data analysis

Each condition was tested at least in three biological repeats. The following parameters were measured on the basis of collected data:

1 $$Surface\,transfer\left(T\right),\%:T=\frac{{log10\,CFU}_{surface,\:\:t}*100}{log10\:{CFU}_{gelatin}\:}$$

where log_10_CFU_surface, t_= log_10_ CFU on the studied surface at timepoint (t) of interest

and log_10_CFU_gelatin_=log_10_ CFU on gelatin before surface transfer

2 $$\begin{aligned}\\&Antimicrobial\,activity (R), log_{10}\,CFU:\:R={{log}_{10}\,CFU}_{control,t}\\&-{{log}_{10}\,CFU}_{test,t}\:\:\:\:\end{aligned}$$

where log_10_CFU_control, t_= log_10_ CFU on the control surface at selected timepoint (t).

and log_10_CFU_test, t_=log_10_ CFU on test surfaces at selected timepoint (t).

3 $$\begin{aligned}\\&Surface\,kill\,over\,time (S), log_{10} CFU:\:S={{log}_{10}CFU}_{test,0}\\&-{CFU}_{test,t}\:\:\:\:\end{aligned}$$

where log_10_ CFU_test,0_= log_10_ CFU on test surfaces immediately after surface touch.

and log_10_CFU_test, t_=log_10_ CFU on test surfaces at timepoint of interest (10 min in this study).

For data comparison, either an unpaired two-tailed t-test or ANOVA combined with Tukey´s multiple comparisons test was used in GraphPad Prism v.10.

### Laboratories participating in interlaboratory validation

Four laboratories participated in interlaboratory testing all of which had previous expertise in antimicrobial susceptibility testing on antimicrobial surfaces: (i) laboratory of Dr. A. Ivask at the Institute of Molecular and Cell Biology at the University of Tartu, Estonia, (ii) laboratory of Dr. Q. Ren from Swiss Federal Laboratories for Materials Science and Technology, (iii) laboratory of Dr. F. Schreiber from the German Federal Institute for Materials Research and Testing, and (iv) laboratory of Dr. D. Willkomm at the Technische Hochschule Lübeck in collaboration with H. Schnaars, Drägerwerk AG & Co. KGaA.

## Results

### Optimization of HydroTouch test

To enable quantitative comparison of microbial transfer and antimicrobial activity across different surfaces using the HydroTouch test [26], we optimized three key aspects: (i) the characteristics of the bacterial inoculum, (ii) the environmental conditions during transfer, and (iii) exposure duration on the test (antimicrobial) surface. The starting microbial inoculum has a significant importance mainly by determining the dynamic range, i.e., the maximal reduction of bacterial cells in the test. The transfer environment may notably affect the transfer efficacy and survival of microorganisms on the test surface. Exposure time determines the survival of microorganisms on non-antimicrobial, negative control surfaces and clearly affects the antimicrobial efficacy of the test surfaces. Sampling at two exposure timepoints allowed us to quantify the antimicrobial effect of a test surface. This is different to the previously described HydroTouch test, in which only one timepoint directly after contact was sampled, allowing only to determine microbial surface transfer (T).

**Fig. 2:**
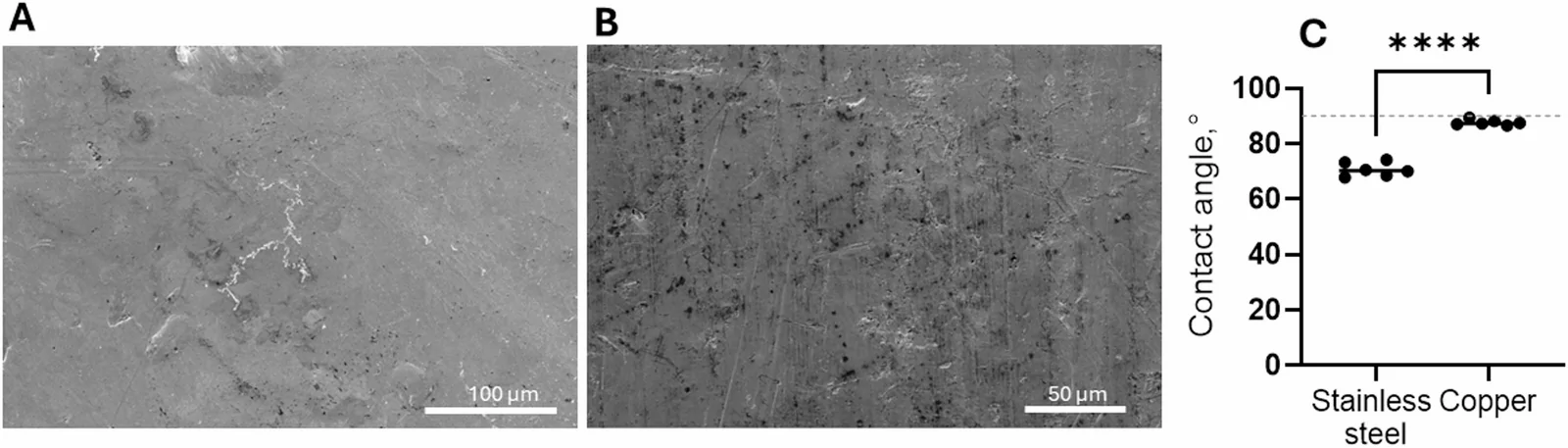
Characteristics of the surfaces used in this study. Scanning electron microscopy images of stainless steel (**A**) and copper (**B**), and the respective water contact angles of both surfaces. In (**A**) and (**B**) scale bar is shown, in (**C**) dashed line indicates 90°, which is used as a separation of hydrophobic and hydrophilic surfaces. **** *p*<0.001 difference between stainless steel and copper according to unpaired two-tailed t-test in GrapPad Prism 10

Surface transfer and antimicrobial activity was measured using copper surfaces and stainless steel as a non-antimicrobial control. SEM images showed that those surfaces were slightly different: while stainless steel was rather smooth (Fig. 2A), clear surface defects were present on copper surface (Fig. 2B). Concurrently also the surface angle of stainless steel and copper was slightly but statistically different (Fig. 2C). Stainless steel showed significantly lower contact angles, i.e. higher hydrophobicity, than copper. This effect could be driven either by intrinsic material properties or differing surface topography as surface defects (e.g., scratches on copper surfaces) may contribute to decreased surface hydrophobicity [29]. Despite of thos small differences, stainless steel was considered as one of the most relevant non-antimicrobial controls for metallic copper.

### The characteristics of bacterial inoculum: number of cells in the test

**Fig. 3:**
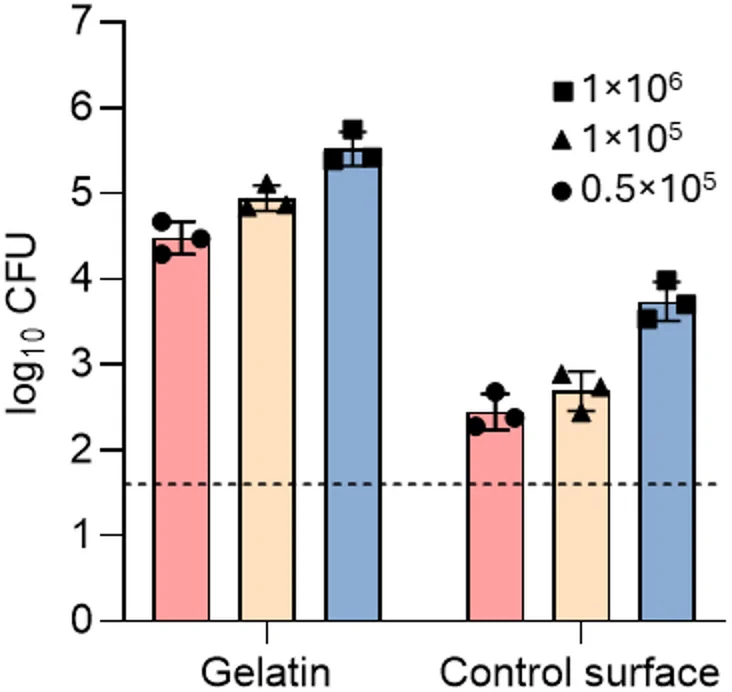
The number of *E. coli* cells on the gelatin stamp and control (stainless steel) surface immediately after touch transfer. Inoculum with cell counts of 5×10^4^, 10^5^ and 10^6^ CFU per gelatin stamp was used. The dashed line represents the limit of detection of the assay. Results of individual measurements, mean (column height) and standard deviation of the measured cell counts from three biological replicates depicted by the individual symbols are shown

In the original HydroTouch test protocol, the number of bacterial cells applied onto the donor surface (gelatin) was 1 × 10^5^ and the transfer efficacy ranged from ~ 1–10%. This would result in the transfer of 10^3^–10^4^ cells onto the surfaces, and considering the dilution effects involved in surface washoff and plating during the regular test procedure 10–100 colonies in final plates could be retrieved. By increasing the plated volume, the number of colonies could be increased by up to 200–2000, however, considering certain losses due to experimental handling and possible death of some of the microorganisms e.g., due to dessication, the number of final colonies retrieved even from non-antimicrobial surfaces may remain barely countable in real test settings. The results of this study showed that indeed, 0.5–1 × 10^5^ cells on the gelatin pad resulted in low transfer to stainless steel as a non-antimicrobial control surface. Increasing the initial cell load to 1 × 10^6^ cells per gelatin pad resulted in retrieving a cell number more than 2 log_10_ above the detection limit (Fig. 3), which was considered sufficient to calculate microbial transfer efficacy, relying on the number of CFUs recovered from gelatin surface instead of comparing CFUs recovered from test surface immediately after stamping or after the incubation time. This increased inoculum concentration can also provide a sufficient range to measure potential reductions over time to quantify potential antimicrobial effects (e.g. of different types of microorganisms transferred in different conditions).

### Exposure duration of bacteria on test surfaces 

**Fig. 4:**
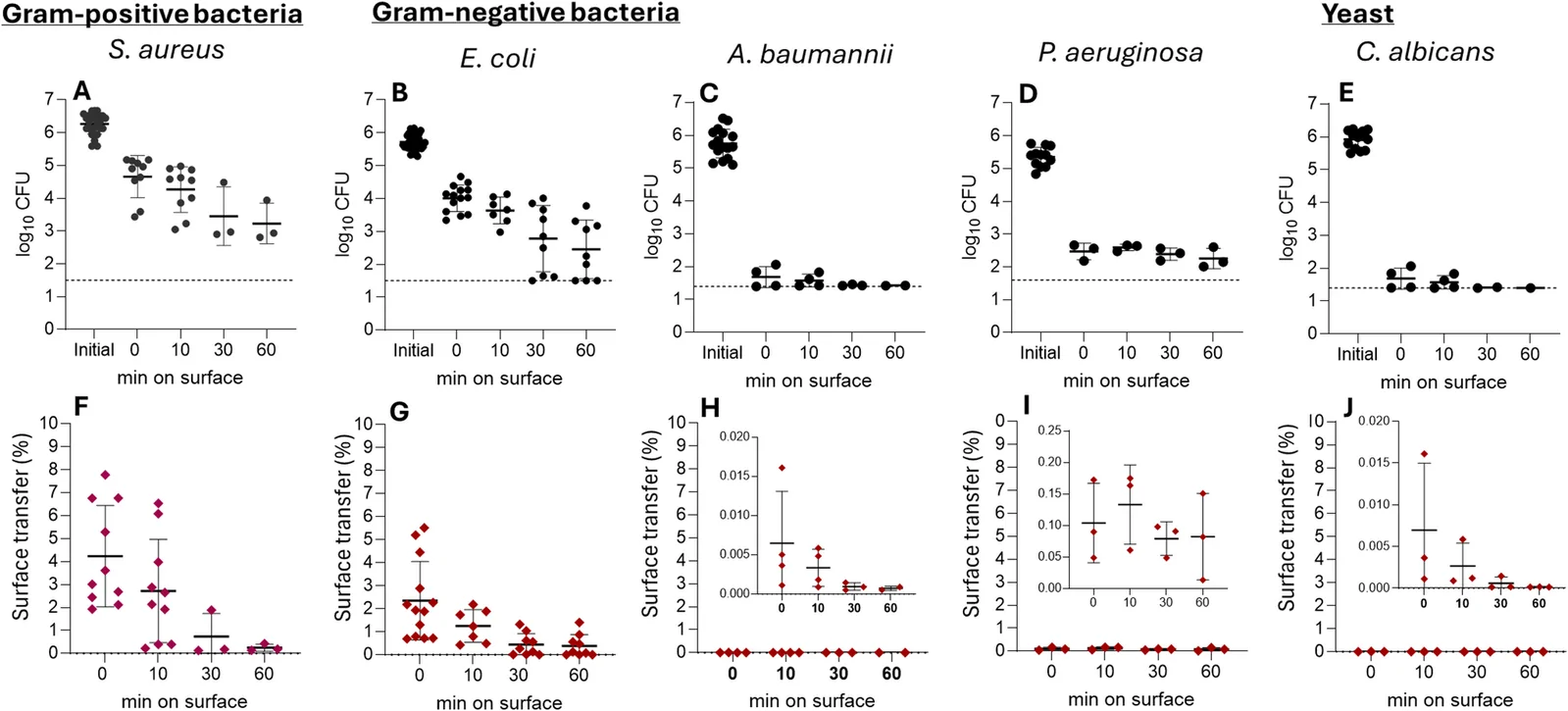
Bacterial and yeast cell numbers on control (stainless steel) surfaces after touch transfer from gelatin. **A**-**E** The initial number of CFU on gelatin (initial) and recovery of CFU from stainless steel directly after stamping and 10, 30 and 60 minutes thereafter. **F**-**J** % surface transfer as calculated for timepoints 0-60 min. Results of individual measurements, mean (horizontal bar) and standard deviation (error bars) of the measured cell counts from minimum three biological replicates (individual circles) are shown

Even if a sufficient amount of bacteria is transferred to the control surface during touch, the bacterial number should stay detectable in order to calculate the antimicrobial activity, i.e., the decrease of CFU on the test surface as compared with non-antimicrobial control at any selected timepoint (Eq. 2). However, it is well known that in semi-dry or dry conditions that are used in the HydroTouch test, bacterial viability decreases. Kaur et al. [19] showed that drying of bacteria in aerosols on stainless steel and glass decreased the number of alive *E. coli* cells by about 4 log_10_ and the number of alive *S. aureus* cells by about 1 log_10_. Maitz et al. [30] showed that drying a bacterial inoculum on a stainless steel surface reduced *S. aureus* by 0.5–1.5 log_10_ and *E. coli* by more than 5 log_10_ within 1 h. Therefore, we determined the survival of a set of bacteria and a yeast in the HydroTouch test on a control surface during 60 min following the gelatine touch (Fig. 4). At 0 min, relatively high levels (~ 1–10%) of *E. coli* and *S. aureus* were transfered to and retrieved from the control surface (Fig. 4F, G), which suggests a rather efficient transfer from gelatine to the surface and is consistent with our previous report [26] indicating transfer rates of 2–3% for *E. coli* and approximately 15% for *S. aureus*. As the after-touch exposure time on the surface increased, progressively fewer bacteria were retrieved from the surface. For both species, a decrease in CFU of around 0.5 log_10_ was observed after 10 min on the surface and an additional 1 log_10_ decrease was detected after 60 min. At the 30 and 60 min timepoints, the CFU count of *E. coli* was very close to the detection limit (Fig. 4B) of the test and therefore, those exposure times were not used in further experiments. Although also *S. aureus* cells gradually died with increasing exposure time on the surface, the amount of surface-retrieved cells remained still well above the limit of detection (LOD) after 60 min of surface exposure to the control surface (Fig. 4A).

In the case of *P. aeruginosa*, *A. baumannii*, and *C. albicans*, the initial surface transfer was already very low. Only 0.1% of *P. aeruginosa* cells were transferred to the surface (Fig. 4I), which is similar to what was observed in our previous study [26]. The surface transfer of *A. baumannii* and yeast was even lower and reached only 0.01% of the initial inoculum on gelatine (Fig. 4H and J). As the cell number decreased even further with exposure time on the surface (Fig. 4C-E) and either remained at or even below LOD (*A. baumannii* and *C. albicans)* or just above LOD (*P. aeruginosa)*, those species were not included in further experiments. The observed species-dependent transfer efficiencies likely reflect different adhesion behaviour of different bacterial strains to the donor surface and detachment during contact with stainless steel. While all the tested organisms are capable of biofilm growth, *P. aeruginosa*, *A. baumannii*, and *C. albicans* are all known to exhibit strong surface-associated and adhesive phenotypes [31], including extracellular polymeric material production and aggregation behavior, which may promote retention on the gelatin donor surface and hence reduce release during transfer. In contrast, *E. coli* and *S. aureus* may interact less strongly with the donor interface under the tested conditions, resulting in more efficient detachment and higher transfer rates. 

**Fig. 5:**
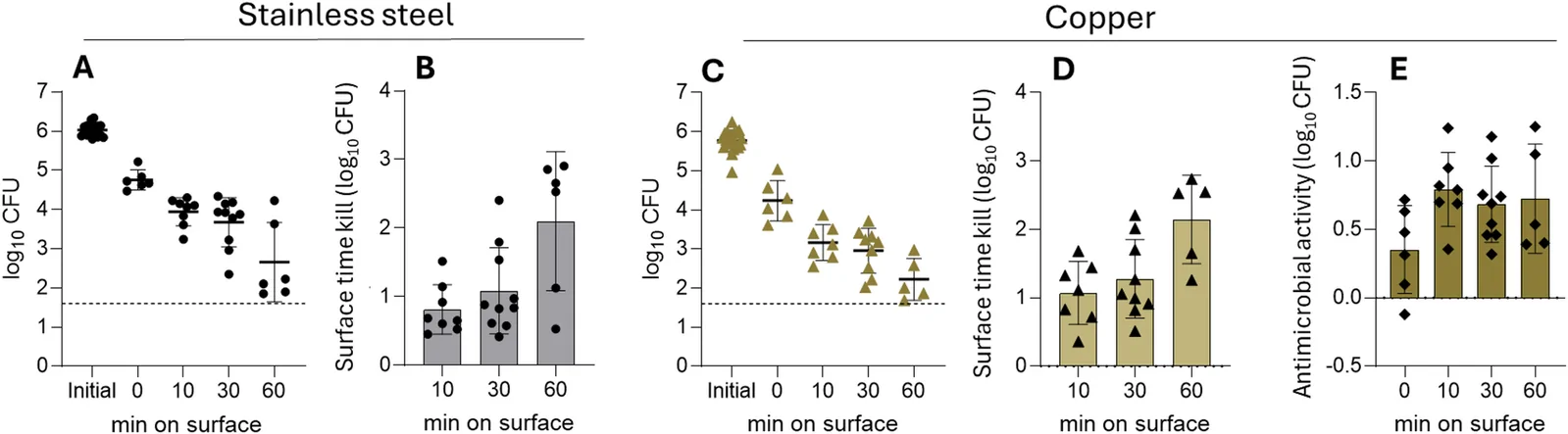
*E. coli *cell number and surface kill over time on control (stainless steel) and copper surfaces after touch transfer. Bacterial number on stainless steel (**A**) or copper (**C**) surfaces after different post-touch exposure times. Surface kill over time on steel (**B**) or copper (**D**) surfaces calculated from data in panel (**A**) and (**C**), respectively. **E** antimicrobial activity of copper surfaces as compared to control was calculated for different post-touch exposure times (CFU on control compared with CFU on copper). Individual values, mean (horizontal bar in (**A**) and (**C**), columns in (**B**, **D**, **E**)) and standard deviation of the measured cell counts from at least 5 biological replicates are shown

The recovery of *E. coli* cells was studied in addition to stainless steel also from copper surfaces. The number of CFUs retrieved from copper surface immediately after touch (0 min exposure) was slightly but statistically lower than that from stainless steel (comparison between Fig. 5A, C and E, *p* < 0.05 in t-test). However, as the difference between CFUs retrieved from stainless steel (around 6 log_10_ CFU) and copper (5.8 log_10_ CFU) was rather small, we suggest that the difference in surface hydrophobicity between steel and copper (Fig. 2) did not play a major role. It is likely that the small CFU decrease on copper may be due to the fast antimicrobial activity of copper. The number of bacteria on copper surface clearly decreased over time (log_10_ CFU retrieved from 0, 10, 30 and 60 min statistically different according to one-way ANOVA, *p* < 0.05), similarly to steel. Starting from 10 min exposure, copper surface reached average antibacterial activity of 0.8 log_10_ CFU (Fig. 5E). Although the CFU on the copper surface continued to decrease after 10 min (Fig. 5D), the calculated antibacterial activity did not increase until the maximal exposure time, 60 min (Fig. 5E). As seen from Fig. 5A, also the CFU count on the control surface decreased and became rather close to the limit of detection. Therefore, the suggested post-touch exposure time that resulted in optimal antimicrobial activity of copper surfaces was 10 min, which was selected as the reference time for the subsequent interlaboratory trial.

### Environmental conditions during touch transfer: the effect of surface soiling 

**Fig. 6:**
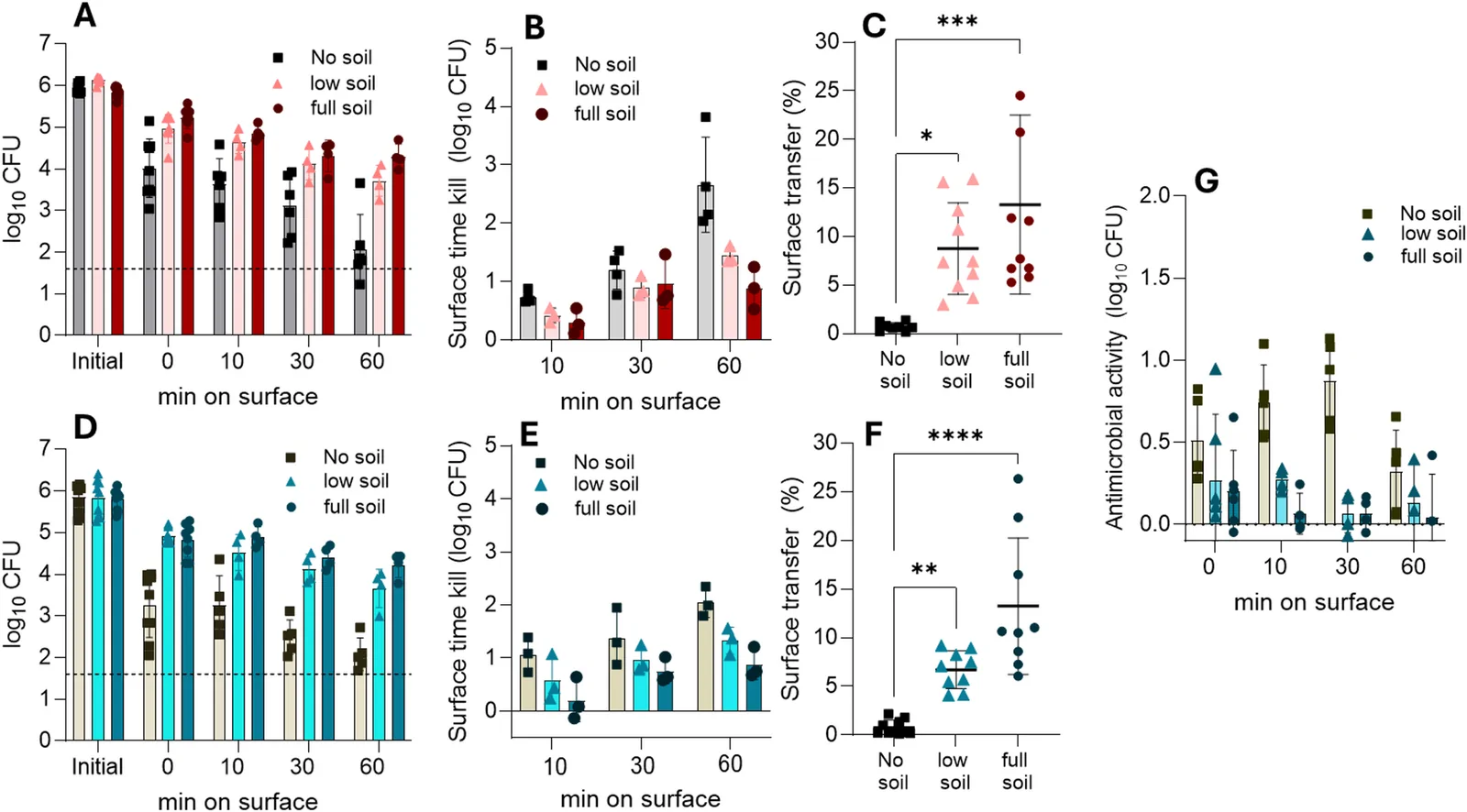
*E. coli* cell number, surface kill over time and surface transfer on control (stainless steel) and copper surfaces after touch transfer with and without artificial soiling. CFU recovered from steel (**A**) or Cu surfaces (**D**) at different post touch transfer timepoints without or with different concentrations of artificial soil (low soil = 3.7 mg ATS/mL full soil = 18 mg ATS/mL). Surface kill over time on either stainless steel (**B**) or Cu (**E**) surfaces. Surface transfer on steel (**C**) or Cu (**F**) surfaces. Antimicrobial activity of copper surface against *E. coli* (**G**). Individual values of at least three biological repeats, average (column height in (**A**, **B**, **D**, **E**, **G**) and horizontal bar in (**C** and **F**)) and standard deviation are shown.* *p* < 0.05, ** *p* < 0.01, *** *p* < 0.005 in one-way ANOVA with Dunnett´s multiple comparisons test

Surface soiling is an important factor influencing microbial survival on surfaces, as well as the efficacy of antimicrobial surfaces. While most touch transfer studies conducted to date have used PBS mineral buffer or organics-poor media, in real-world conditions, organic residues (soil) are always present on surfaces. To account for the impact of surface soiling on bacterial surface transfer and surface antimicrobial properties, an artificial soiling (ATS) mixture was used that, according to the producer, is simulating human body liquid-based soiling (e.g., on medical devices) and has been used for cleaning validation and verification studies. Two concentrations of the soiling mixture were used in the study: the concentration suggested by the producer, which is a representative of worst-case soiling scenario (18 mg ATS/mL), and a 5 times lower concentration (3.7 mg ATS/mL) similarly to a previous study with this and other soiling agents [27]. In the presence of soiling, the surface transfer both onto stainless steel as well as onto copper was significantly higher (*p* < 0.01, one-way ANOVA with Dunnett´s multiple comparsions test; Fig. 6C and F). The same can be evidenced from Fig. 6A and D which shows gradually increasing log_10_ CFU on both, steel and copper surfaces with increasing soil load. While without soiling, surface transfer was ~ 0.5%, then with full soil the transfer to steel was 11 ± 4% and to copper 7 ± 2%. It is however also important to note that the presence of soiling significantly reduced antibacterial activity of copper surface (Fig. 6G). While without soiling, copper surface decreases surface bacterial count by ~ 0.7 to 1 log_10_ CFU within 10–30 min, with low or full soiling (3.7–18 mg ATS/mL) the antimicrobial activity almost disappears. The same effect can be seen by increased log CFU counts on copper surface in the presence of soiling (Fig. 6D) and is mostly likely due to the protective effect of the organic components from copper toxicity but also due to enhanced water retention in the presence of organic molecules as has been earlier demonstrated by Kaur et al. [19]. Although surface soiling is highly relevant from the point of view or real life use of antimicrobial surfaces, the following interlaboratory comparison of HydroTouch test was carried out without surface soiling. This choice was justified by almost inexistent antimicrobial activity of copper surfaces in the presence of soiling, which would not enable any quantitative comparisons.

### Interlaboratory comparison of the performance of HydroTouch in evaluating microbial transfer and antimicrobial properties of surfaces

We further utilized the optimized HydroTouch test parameters to carry out an interlaboratory comparison of the performance of the test as a tool to evaluate microbial surface transfer and antimicrobial activity under semi-dry conditions. The four participating laboratories performed the HydroTouch testing following a standardized protocol, and standardized laboratory consumables, control and test surfaces. The cotton swabs used for cell recovery from surfaces were distributed between the participating labs and a video was shared to standardize the swabbing procedure. The gelatin cutting device and gelatin holders, used in touch transfer, were shared between labs 1–3. Laboratories 1–3 shared additional practical details regarding the storage of gelatin pads at 4 °C before the application of bacterial inoculum, and carrying out the post-touch incubation of the surface in a closed box. Despite of a standardized protocol, relative air humidity (RH) and exposure temperature slightly varied between the participating laboratories. Specifically, the RH value in labs 1, 2, 3 and 4 were 36–55%, 32%, 50–60%, and 31–50%, respectively, and the temperatures in the respective labs were 22, 26, 24 and 22 °C. Also the box used for post-touch accommodation of the gelatin pads differed between labs, having volume of 0.6 L in labs 3 and 4, 1.1–3 L in lab 1 and 5.8 L in lab 2. The differing size of the storage box may have affected the local air humidity and with this having an effect on bacterial dessication on surfaces.

**Fig. 7:**
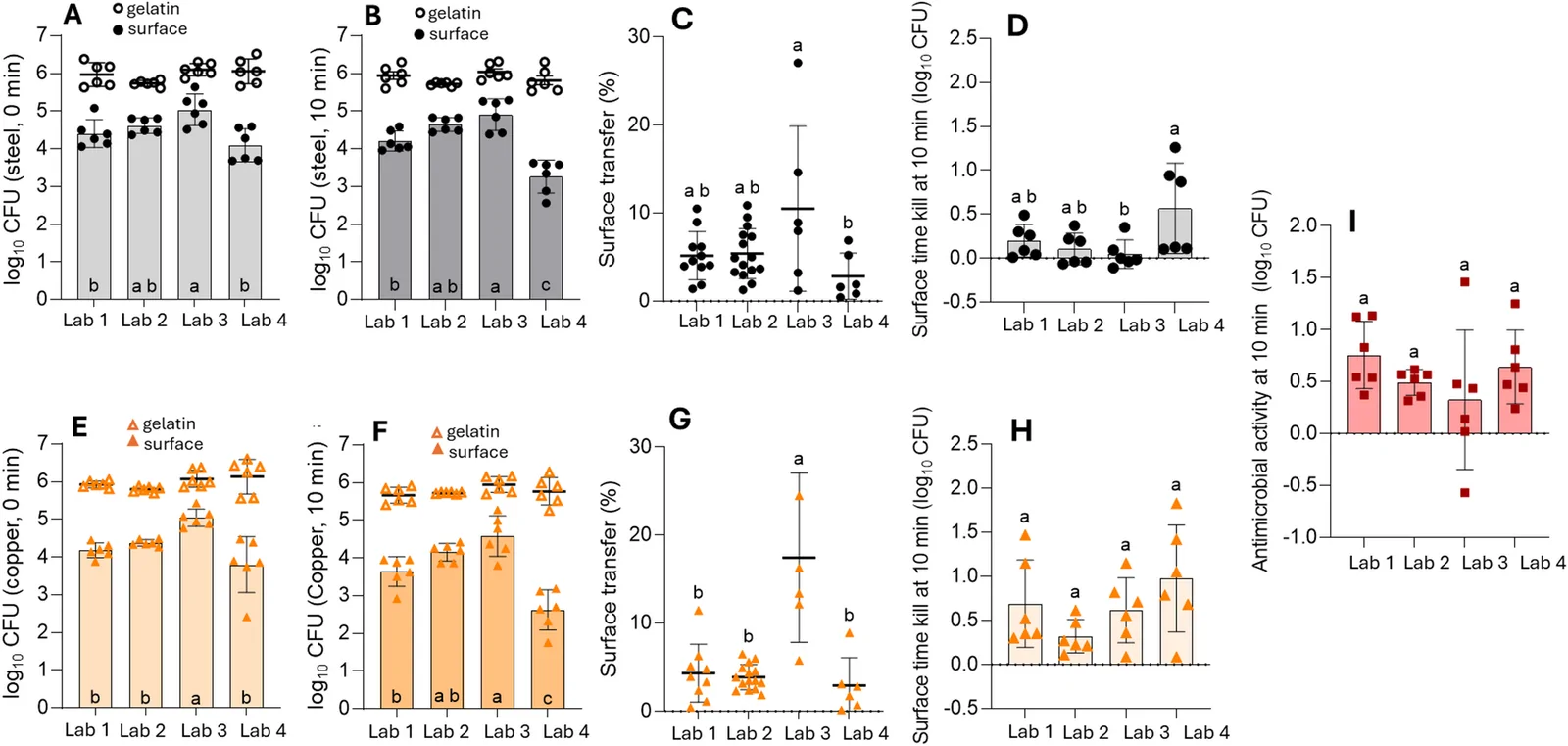
Comparison of the results of interlaboratory study. **A**, **B**, **E**, **F**: closed symbols and columns indicate individual and average (horizontal line or column height) *E. coli* log_10_ CFU retrieved from stainless steel (**A**) or copper (**E**) immediately after touch, or *E. coli* log_10_ CFU retrieved from stainless steel (**B**) or copper (**F**) at 10 min of post-touch exposure. Open symbols indicate CFU retrieved from respective gelatin surfaces. Surface transfer to stainless steel (**C**) or copper (**G**). Surface kill over time on steel (**D**) or copper (**H**) at 10 min post-touch exposure. Standard deviation refers to at least six replicates. Antimicrobial activity of copper surface 10 min after touch (**I**). On (**C**, **D**, **I**, **G**, **H**) individual measurements, mean (horizontal line or column height) with standard deviation are shown (minimum six replicates). Multiple pairwise comparisons between labs are expressed as letters indicating statistically significant (*p* ≤ 0.05) differences (similar letter shows no significant difference, different letters indicate *p* ≤ 0.05 diffrence between labs)

Bacterial counts retrieved from stainless steel and copper surfaces immediately after the finger-mimetic touch showed some interlaboratory variability (Fig. 7A and D). As a general trend, laboratory 4 measured the lowest amount of CFUs while laboratory 3 measured the highest number of CFUs whereas similar results were obtained by laboratories 1 and 2. Due to these variations also the calculated surface transfer of *E. coli* cells varied between the labs (Fig. 7C and D) from gelatin to recipient surfaces varied. The lowest mean transfer to steel was observed in lab 4 (2.8%) and the highest in lab 3 (10.5%). Surface transfer in labs 3 and 4 was statistically different (*p* < 0.05, Fig. 7C) but these were not significantly different from labs 1 and 2. In addition, the transfer onto copper surfaces followed the same trend; the lowest mean transfer in lab 4 (2.9%) and the highest in lab 3 (17.4%). These differences could be due to differing RH values as described above. Laboratory 3 had the highest RH during the test and preserved the highest amount of moisture inside the gelatin. At the same time, lab 4 did not use a closed container during inoculum preparation and post-touch surface exposure, and experienced the highest water loss. This suggests that the humidity of the environment during surface exposure plays a major role in surface transfer and survival of bacteria on surfaces.

After 10 min post-touch exposure on surfaces, all the labs observed some level of decrease in CFU counts (comparison between Fig. 7A and B, E and F). On steel, the highest decrease between 0 and 10 min of exposure (0.8 log_10_ CFU corresponding to surface kill over time on Fig. 7D) was reported by lab 4 and we suggest that this was due to the sample incubation not carried out in a closed container, as discussed above. Labs 2 and 3 showed the lowest CFU decrease on the steel surface between 0 and 10 min exposure and in the case of lab 1, the decrease in *E. coli* log_10_ CFU was ~ 0.3 (Fig. 7D). The CFU decrease on copper between 0 and 10 min of exposure was always higher than the decrease on steel (compare surface kill over times on Fig. 7D and H). Although there was no statistical difference between surface kill on copper in different labs, the highest mean kill was observed for laboratory 4 (mean 1.2 log_10_ CFU decrease) followed by laboratories 1, 3, and 2 (Fig. 7H). The calculated antimicrobial effect of copper surface after 10 min post-transfer ranged from 0.3 to 0.75 log_10_ CFU (Fig. 7I) and showed some variability within and between the labs but no statistical differences between labs could be revealed.

## Discussion

In this study we optimized and validated the HydroTouch method for assessing the microbial transfer and antimicrobial efficacy of surfaces. The test involves the transfer of a specified number of microbial cells from a finger-mimetic gelatin surface to a recipient (test) surface during a short, 10 s touch, and subsequent exposure of the transferred cells to the test surface. Specifically, the test enables the calculation of two parameters: efficacy of transfer from gelatin to the test surface (fingertip-mimicking surface transfer), and antimicrobial activity of the test surface (expressed as CFU reduction) during a specified incubation time.

Compared with the classical regulatory efficacy requirement for antimicrobial surfaces, that is a 3 log_10_ reduction within 60 min according to the EU Biocidal Products Regulation guidance (European Union Biocidal Products Regulation [16]), the antimicrobial activity values obtained with the HydroTouch test even for the benchmark copper surface appear lower. However, the regulatory requirement is based on standard liquid-based antimicrobial efficacy tests such as ISO 22196:2011, and HydroTouch test conditions differ fundamentally from those in terms of bacterial transfer (via touch) and humidity of exposure environment (semi-dry to dry). It is well known that lowering the humidity of the test environment and adding organic soiling decreases significantly the antibacterial activity of surfaces [19], and 3 log_10_ CFU reduction is almost impossible to achieve in such conditions. For example, also in real-life studies involving copper surface the maximal microbial count reduction has been ~ 60% [32], which suggests that for real-life scenarios, a realistic expectation should be set, if real-life use imitating tests are carried out. The antibacterial effect of copper surfaces measured during 10 min exposure in this study ranged between 0.3 and 0.75 log_10_ CFU decrease. Considering that copper has been regarded as one of the most efficient antimicrobial surfaces, then this level of antibacterial activity is probably one of the highest that can be achieved in HydroTouch test and antibacterial efficacy as high as 3 log_10_ can not be expected and alternative cut-off values should be considered for antimicrobial claims based on such test methods.

An important aspect that determines the performance of an antimicrobial test is its maximal working range, i.e., the maximal level of log_10_ decrease that can be reliably detected. During optimization of the HydroTouch test, the initial CFU count in the test was increased to ensure that at least a 2 log_10_ decrease after surface touch could be measured. However, the optimized inoculum was suitable for use on metal surfaces like steel and copper but it is possible that for other surface types, for which the microbial transfer is lower than that measured in case of the metal surfaces (minimally 0.15%), the inoculum size should be further increased.

In addition to the unknowns related with microbial transfer efficacy, in touch transfer tests prolonged incubation times can lead to confounding effects, primarily microbial death due to desiccation rather than the antimicrobial activity of the surface itself. This issue is particularly pronounced for strains that are highly susceptible to drying, potentially leading to an overestimation of antimicrobial efficacy or misinterpretation of results. Therefore, dessication-induced death of bacteria on test surfaces can be considered as one of the limitations of the method.

As mentioned above, there is currently no standardized guidance for defining exposure times in dry or semi-dry contact antimicrobial tests. Therefore, we here established an exposure time condition to balance a sufficient contact duration to detect antimicrobial activity while minimizing the confounding effects of desiccation. The optimal time that suited the *E. coli* and *S. aureus* strains used was 10 min, as longer exposure times, such as 30 and 60 min, resulted in too high levels of bacterial death, and inability to measure any further antimicrobial effects. It should be mentioned that other tested bacteria, *P. aeruginosa* and *A. baumannii* as well as yeast *C. albicans* were either highly susceptible to desiccation or were transferred to metal surfaces with such a low efficacy that these could not be used in antibacterial testing of the surfaces.

Our results further demonstrated that bacterial transfer does not only depend on the type of surface and microbial species but also on the presence of soiling on the donor surface. In the presence of organic components on surfaces, the microbial transfer was significantly increased and also the dessication-dependent decrease of bacterial cells was lower, probably due to retention of water by the organic compounds. However it was also clear that the presence of organic soil also reduced the activity of otherwise antimicrobial copper surface: the ~ 1 log_10_ antibacterial activity observed when bacteria were touch transferred on copper in very diluted medium decreased to literally zero when full artificial soil was included into the touching environment. This result indicates that the HydroTouch test format resembles the real-life transfer pathway for microbial cells between surfaces, and that difficult to control parameters, such as surface soiling, may affect the final antimicrobial activity result.

Given the above, one of the important conclusions of this study is that in real-life settings 1 log_10_ reduction could be even sufficient for an antimicrobial claim. Firstly, we show how in semi-dry environments, clinically relevant pathogenic species get transferred below 10% of the initial cell density (that is already 1 log_10_ reduction in the transfer process), and desiccation within 60 min determines further decrease of at least another log_10_ reduction (effect predominantly observable with *C. albicans*, *A. baumannii*, *P. aeruginosa*). This implies that in reality, an antimicrobial surface capable of providing a 1 log_10_ reduction within the defined time period combined with limited transfer and desiccation already exert a 3 log_10_ reduction over the original contamination source. The relevance of our results are corroborated by similar studies where implementation of real-life conditions showed reduction of bacteria on copper by approximately 1 log_10_ only [33]. Secondly, we show that in the same conditions, soiling heavily impacts the antibacterial activity of copper surface and as it is also known to affect the efficacy of other types of antimicrobial surfaces [19], this parameter should be included in both testing guidelines as well as in requirements for antimicrobial activity assessment.

In order to evaluate the validity of HydroTouch test results and robustness of the test procedure, an interlaboratory comparison was carried out. To ensure comparability between the labs participating to the interlaboratory study, clear procedural details were agreed upon. Indeed, our results showed that in case of a deviation from the agreed procedure, slight differences in results were measured. One important parameter that was found to affect the microbial transfer between surfaces was relative air humidity. Clearly, the lab where RH during the test was the highest showed the highest bacterial transfer from the mimetic finger to metal surfaces, whereas the lab which carried out surface exposure at ambient conditions without specific containment to preserve humidity, showed the lowest transfer. Therefore, for higher reproducibility and decreased variability it is advised to carry out surface transfer tests in more precisely controlled humidity conditions, e.g., in a humidity controlled climate chamber. Moreover, our experiments showed that the „sticking“ properties of gelatine pads are temperature and humidity dependent and for better standardization, their storage conditions should be well defined. For example, in this study we suggested to store the pads at 4 °C whenever possible. One additionally important detail concerned swabs and swabbing techniques used to recover cells from surfaces, which may significantly influence the test results [34]. In our interlaboratory testing, all laboratories used cosmetic cotton-tipped swabs and received a video training for the swabbing method. Despite those standardizations, there were significant differences between surface-retrieved CFU counts between the participating labs, which were likely caused by other undescribed details. This result is not surprising as even for the benchmark method for surfaces antimicrobial testing, the ISO 22196:2011 different labs have received varying results in interlaboratory comparisons, especially in the case of intermediate-level antimicrobial surfaces [17]. In this previous article the most important factors that determined the antimicrobial effect were (i) incubation time, (ii) starting concentration of the bacteria as well as (iii) physiological state of the bacteria (stationary or exponential phase of growth), and (iv) concentration of the nutrients, plus other practical details. Even if most of those factors were suggested to keep constant in this study, they did not completely remove the interlaboratory variations. However, our results showed that even when the number of bacterial cells transferred between surfaces may differ between the labs, the antibacterial effect of copper surfaces was still measureable: although there was some decrease in bacteria viability on steel surfaces due to dessication, on copper surfaces the decrease was always higher. Additionally, this reduction on steel surfaces being always less than 1 log_10_, supports the fact that the result repeated from lab to lab were reproducible.

Overall, our study highlights the potential of the HydroTouch test for widespread use and also highlights protocol details that require further improvement and standardization. For example, differences in test and control materials could be minimized by using surfaces with similar surface finish, achieved through the same polishing treatment; this would lead to more comparable topologies of the gelatin-stamped surfaces, with a more comparable transfer percentage. Additionally, a more strict definition of incubation conditions should be defined to control the RH of the surfaces during the incubation time. These could be (i) a defined time and temperature condition of gelatine storage prior to its inoculation (which would entail a more controlled initial water content and temperature of the gelatin pad), (ii) a defined volume and temperature incubation of the closed container with the stamped surfaces during the chosen exposure time, and the potential usage of humidifier within it (which would entail a narrower and more predictable humidity range), and (iii) a specific bacterial strain more resistant to desiccation for extended incubation times. Altogether the above conditions might be tailored to reflect the real-life scenario that the test is aimed at investigating, which is a critical point for evaluating the efficacy of any antimicrobial material that is intended for a specific use. Clearly, the HydroTouch test offers a novel tool for evaluating the efficacy of antimicrobial touch surfaces under real-life use relevant conditions and could therefore be used to support the development and selection of more effective surfaces to reduce microbial transfer in critical settings.

## Data Availability

All the datasets are available upon request from corresponding authors.
